# Efficacy and Safety of Woohwangchungsimwon Combined with Donepezil in Behavioral and Psychological Symptoms of Dementia in Patients with Probable Alzheimer’s Disease: Study Protocol for a Randomized Controlled Trial

**DOI:** 10.3390/healthcare11142036

**Published:** 2023-07-16

**Authors:** Man Gi Kim, Mi-Suk Hyun, Sang Gu Park, Eun Cho, Jinsik Kim, Hyung-Kyoon Choi, Kyung-Lak Son, Chi-Yeon Lim, Kwang Ki Kim, Byung Soo Koo

**Affiliations:** 1 Department of Oriental Neuropsychiatry, Dongguk University Ilsan Oriental Hospital, Goyang 10326, Republic of Korea; 2 Department of Oriental Neuropsychiatry, Graduate School of Dongguk University, Seoul 04620, Republic of Korea; 3 Department of Nursing, Kyungdong University, Wonju 26495, Republic of Korea; 4 Department of Neurology, Dongguk University Ilsan Hospital, Goyang 10326, Republic of Korea; 5 College of Pharmacy, Sookmyung Women’s University, Seoul 04310, Republic of Korea; 6 Department of Medical Biotechnology, College of Life Science and Biotechnology, Dongguk University, Seoul 04620, Republic of Korea; 7 College of Pharmacy, Chung-Ang University, Seoul 06974, Republic of Korea; 8 Department of Psychiatry, Dongguk University Ilsan Hospital, Goyang 10326, Republic of Korea; 9 Department of Biostatistics, College of Medicine, Dongguk University, Goyang 10326, Republic of Korea

**Keywords:** Alzheimer’s disease, behavioral and psychological symptoms of dementia, herbal medicine, randomized controlled trial, woohwangchungsimwon

## Abstract

Behavioral and psychological symptoms of dementia are a major factor in the burden of care and medical expenses. Conventional pharmacological treatments do not exert a distinct effect on the benefits versus the risks. The herbal medicine woohwangchungsimwon is frequently prescribed for neuropsychiatric disorders. An effect of woohwangchungsimwon on behavioral and psychological symptoms of dementia has been previously reported; however, no clinical studies have been conducted. We aim to evaluate the efficacy and safety of woohwangchungsimwon combined with donepezil for alleviating these symptoms in probable Alzheimer’s disease. In this randomized, assessor-blinded, parallel-group clinical trial, 74 participants with probable Alzheimer’s disease will be divided via block randomization into a woohwangchungsimwon + donepezil combination group (n = 37) or a donepezil single group (n = 37). Participants will include patients under donepezil treatment for at least a month. We will perform the study for 24 weeks. The Neuro-Psychiatric Inventory subscale scores will be the primary outcome. Secondary outcomes will include cognitive function, dementia severity, physical function, quality of life, depression, anxiety, and insomnia. For safety evaluation, we will assess adverse reactions, measure vital signs, and conduct laboratory tests. This is the first trial aiming to confirm the efficacy and safety of woohwangchungsimwon combined with donepezil for alleviating behavioral and psychological symptoms of dementia. Its findings could provide a basis for their co-administration to control these symptoms in probable Alzheimer’s disease.

## 1. Introduction

The prevalence of dementia is increasing with an aging population and is expected to reach 113 million by 2050 [1]. Alzheimer’s disease (AD) is a progressive age-related neurodegenerative disease that is the most common cause of dementia and is principally characterized by cognitive impairment and neuropsychiatric symptoms (termed behavioral and psychological symptoms of dementia; BPSD) [2]. More than 90% of patients with dementia have at least one BPSD [3]. BPSD is a significant factor in the burden of care, increasing medical costs and early institutionalization [4], and its occurrence can accelerate cognitive decline in AD [5]. Hence, BPSD is an important target in treating AD and cognitive decline.

In clinical settings, antipsychotic drugs and antidepressants are commonly used for BPSD treatment. Antipsychotics exert an immediate effect but may exhibit extrapyramidal side effects, tardive dyskinesia, and increased mortality [4]. Antidepressants may be used for depression, but their long-term efficacy is unclear; moreover, they can exert side effects, such as hyponatremia [6]. The existing drug treatments do not address all aspects of BPSD and may potentially cause serious side effects. This warrants novel alternatives owing to the limitations of existing treatments.

In East Asian countries, herbal medicines have been used for thousands of years to treat memory loss, including dementia [7]. Recently, herbs with anti-acetylcholinesterase activity, anti-amyloid beta aggregation activity, and antioxidant effects, which are closely related to the pathology of dementia, have been identified in in vivo and in vitro models [8]. Furthermore, herbal medicines are being used to control BPSD. A meta-analysis conducted in 2017 suggested Yokukan-san and EGb761 as promising therapeutic agents to improve BPSD and cognitive function in patients with dementia [9].

Woohwangchungsimwon (WCW) is the most widely used herbal medicine for mental and neurological diseases in oriental medicine, with 20 million doses being administered annually in Korea [10]. A previous study that analyzed the database of more than 1000 oriental medicine books identified 12 references that cited WCW as a prescription for dementia and cognitive impairment [11]. WCW is used widely for palpitations, mental anxiety, and autonomic ataxia [12], and it is approved by the Korean Ministry of Food and Drug Safety for indications such as mental anxiety, palpitations, and mental confusion. It activates the parasympathetic nervous system [13] and is effective against palpitation and anxiety [14]. In experimental studies, WCW improved depressive-like behavioral symptoms in a mouse model [15], besides exerting anti-acetylcholine and anti-barium chloride activities [16]. An epidemiological study in Korea suggested that depression, apathy, and anxiety were the most common BPSD in early-stage AD [17]. Previous clinical and preclinical results highlight the effect of WCW on depression and anxiety; thus, clinicians can expect the possibility of using BPSD in early AD. In contrast, no clinical studies have examined the efficacy of WCW in alleviating BPSD.

Recently, combination therapies that increase the efficacy of donepezil have been emerging in dementia treatment, and herbal medicines have been proposed as one of the treatments for combination use [18]. Donepezil is one of the FDA-approved dementia drugs that can inhibit acetylcholinesterase. According to Korean health insurance data, donepezil was the most commonly prescribed drug among dementia drugs, such as rivastigmine, galantamine, and memantine [19]. Despite decelerating the rate of cognitive decline, it exerts a relatively weak effect on BPSD [20]. Moreover, it leads to a risk of side effects, such as nausea, loss of appetite, and sleep disturbance, owing to increased acetylcholine levels [20,21]. The combination of herbal medicine and donepezil is more effective in dementia patients than donepezil monotherapy [22,23,24].

Therefore, we intend to examine if the additional administration of WCW can relieve BSPD in patients under donepezil treatment, the most commonly prescribed dementia drug.

We present the following article/case in accordance with the Standard Protocol Items: Recommendations for Interventional Trials (SPIRIT) reporting checklist [25].

## 2. Experimental Design

### 2.1. Objective

The primary aim of this study is to determine if the combination of WCW and donepezil is superior to donepezil alone in alleviating BPSD in patients with probable AD.

### 2.2. Study Design and Recruitment

**Figure 1:**
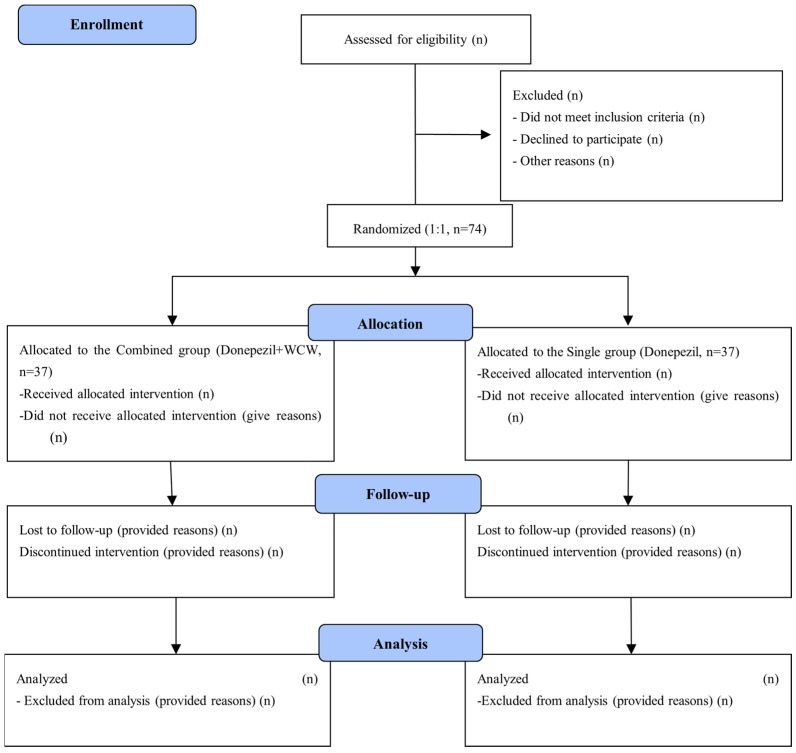
Diagram of the study flow. This figure depicts a summary of the progress of the clinical trial. fMRI, Functional Magnetic Resonance Imaging.

This randomized, assessor-blinded, parallel-group trial will be conducted at the Korean Medical Hospital of the Dongguk University and Medical Hospital of the Dongguk University in Ilsan, Republic of Korea, from December 2020 to February 2024. Figure 1 outlines the study process. Seventy-four participants diagnosed with mild probable AD will be recruited. According to the NINCDS-ADRDA, the diagnosis of AD is divided into definite, probable, and possible AD. For definite AD, the diagnostic pathology of AD must be confirmed at autopsy, but it is difficult to obtain consent from patients in actual clinical settings, and there are various considerations, such as time and cost. Therefore, this study plans to recruit patients with probable AD, which is the most accurate diagnosis within the limit that can be made clinically without pathological confirmation.

The recruitment of participants was planned using a combination of in-hospital and out-of-hospital advertisements. Posters containing clinical trial information will be displayed within the hospital; kiosks, banners, and leaflets will also be used as recruitment tools. In addition to newspapers, advertisements on buses and subway carriages are also planned for outside advertising. Social media and professional platforms to recruit participants were also considered, but it was ultimately concluded that their effect would be negligible given that most potential participants are expected to belong to the elderly population. In addition, the research team is operating a Western–Oriental joint brain health clinic and dementia-related research is actively underway. This is expected to appeal to additional potential participants.

The participants will be divided into two groups in a 1:1 ratio, namely the combination group (Donepezil + WCW) and the single group (Donepezil). The combination group will be administered WCW (Kwangdong Pharmaceutical Co., Seoul, Republic of Korea, Table 1), one pill/time, once/day 30 min following dinner, and donepezil. The single group will be only administered donepezil.

All participants must be completely informed of the study purpose and sign a consent form provided by the Clinical Research Coordinator. Participants who meet the inclusion criteria will be provided with identification codes. Each participant will be screened at visit 1, followed by four additional visits at intervals of 2 months ranging from visit 2 to visit 5. The interval between visits 1 and 2 will be less than one week. The trial will be conducted for 24 weeks. Table 2 presents the schedule for the follow-up visits. We will investigate the presence of adverse reactions at visits 3, 4, and 5.

### 2.3. Eligibility Criteria

We will include the participants who fulfill the inclusion criteria, as depicted in Table 3.

### 2.4. Randomization

A block randomization method using a predetermined block size will ensure a balanced allocation of the participants to each group. A statistician will perform the randomization using the SAS statistical software (version 9.4, SAS Institute, Cary, NC, USA), thus allowing a 1:1 assignment between the groups. The generated randomization table will be delivered to the code manager. In case of an emergency, the code will only be released to the relevant personnel. All procedures will be performed in accordance with the standard operating guidelines for random code release.

### 2.5. Blinding

This study will not have a placebo for WCW. The clinical trial drug is formulated as a pill, and the exterior is covered with gold leaf, which exerts a soothing effect. A placebo should be physically identical to the clinical trial drug but without a pharmacological activity [26]. However, in the case of WCW, it is difficult to imitate the distinctive taste, color, and smell upon maintaining the shape. By contrast, it is difficult to replace the soothing effect of the gold leaf upon changing the shape. Therefore, a double-blind trial is practically impossible, and the study will be single and partially blind. Hence, the evaluators will be blinded to remove the potential bias.

## 3. Detailed Procedure

### 3.1. Intervention

Patients under treatment with donepezil (5 mg), one tablet/time, and once/day 30 min following breakfast for at least one month will be included in this study. They will be divided into two groups as follows: the combination group (Donepezil + WCW) and the single group (Donepezil). The combination group (intervention group) will be administered WCW (Kwangdong Pharmaceutical Co., Seoul, Republic of Korea), one pill/time, once/day 30 min following dinner, and continue to use donepezil for 24 weeks. The single group (control group) will use only donepezil for 24 weeks.

### 3.2. Outcomes

#### 3.2.1. Primary Outcome

The primary outcome will be a change in the Neuro-Psychiatry Inventory (NPI) total score from the baseline to week 24. The NPI is the most commonly used questionnaire to assess BPSD [27], and it includes 12 subscales: delusions, hallucinations, agitation, irritability, depression, anxiety, euphoria, disinhibition, aberrant motor behavior, apathy, sleeping disorder, and eating disorder. For each subscale, the score is calculated as “severity (1–3) × frequency (1–4)”, with a maximum total score of 144 [28]. A clinical psychology practitioner with over 10 years of experience in the Department of Neurology will conduct NPI. Clinical psychology practitioners have been recognized in Korea as experts in administering neuropsychological tests, including SNSB, and have been specifically mentioned as qualified to perform NPI by the dementia relief center project in Republic of Korea.

#### 3.2.2. Secondary Outcomes

We will use several scales to examine cognitive function, dementia severity, physical function, quality of life, depression, anxiety, and sleep quality.

Cognitive function: The Korean version of the Mini-Mental State Examination (K-MMSE) is a reliable and validated cognitive function screening test. Its cut-off score is usually 24 but can vary depending on age and educational level [29]. The Seoul Neuropsychological Test is a comprehensive neuropsychological test for cognitive function in Korea. It enables an in-depth evaluation of each domain [30]. The Alzheimer’s Disease Assessment Scale Cognitive Behavior Section (ADAS-cog) is widely used to assess cognitive function, particularly in clinical trials [31]. The Revised Memory and Behavior Problem Checklist evaluates memory and behavioral problems caused by a cognitive decline when family caregivers care for the elderly. Each question is answered according to a five-point scale [32].

Dementia severity measurement and physical function: The Clinical Dementia Rating (CDR) evaluates a patient’s overall cognitive and social function through interviews with patients and caregivers. Each scale is scored from 0 to 5 (0: no dementia, 0.5: questionable, 1: mild, 2: moderate, 3: severe, 4: profound, and 5: terminal). The maximum total score or the Sum of Boxes is 30 [33]. The Global Deterioration Scale, along with the CDR, measures the severity of dementia. It evaluates the clinical picture and severity of dementia in these patients in seven stages (1–2: normal, 3: compatible, and 4–7: mild to severe AD) [34]. The Korean-style daily life activity tool is a questionnaire that evaluates the daily life performance. The score is calculated from 0 to 1, and the cut-off score is 0.4 [35].

Quality of life: The Geriatric Quality of Life-Dementia Scale is a self-reported questionnaire comprising 15 questions that measures the quality of life of older adults with dementia. Each question is rated on a scale of 1 to 4, and the total score ranges from 15 to 60 [36]. The EuroQol-5 Dimension (EQ-5D) evaluates the quality of life regarding health problems [37].

Depression, anxiety, and sleep quality: The Geriatric Depression Scale is widely recognized as an evaluation tool to screen the elderly for depression. The total score is 15 (classified as 0–4: normal, 5–8: mild depression, 9–11: moderate depression, and 12–15: severe depression) [38]. The Spielberger State-Trait Anxiety Inventory is a self-reported questionnaire that measures the following two dimensions of anxiety: (i) state anxiety, which refers to the anxiety felt in a particular situation; (ii) trait anxiety, which refers to the anxiety experienced on a regular basis [39]. The Pittsburgh Sleep Quality Index is a measure of the quality of sleep [40].

### 3.3. Additional Analysis

#### 3.3.1. Functional Magnetic Resonance Imaging

Functional Magnetic Resonance Imaging (fMRI) imaging can analyze regional cerebral blood flow distribution and potential correlations between regions [17]. Drug treatment has been reported to normalize functional brain connectivity and can be confirmed by fMRI [41]. Moreover, studies have also been conducted on oriental medicine [42]. This study will also examine the changes in brain functional connectivity before and after drug treatment in patients with probable AD.

In the clinical study, 20 participants (10 each in the combination group and single group) will undergo fMRI imaging using 3T MRI scanners (Skyra, Siemens, Germany) on a first-come, first-served basis. We will obtain functional images with the following parameters: repetition time = 2000 ms, echo time = 30 ms, flip angle = 90°, voxel size = 3 × 3 × 4.5 mm^3^, and slice number = 31. Images will be captured before initiating the trial and following drug administration at 24 weeks. The participants will be instructed not to sleep with their eyes closed during imaging.

#### 3.3.2. Economic Evaluation

This study will compare the economic effectiveness outcomes between the donepezil + WCW group and the donepezil-alone group based on the trial data. The incremental cost will be evaluated over the incremental clinical effectiveness, such as the NPI and K-MMSE, and the quality-adjusted life years measured using the EQ-5D values.

Most relevant cost information will be obtained from the clinical trial questionnaire. Additional data required to estimate the average indirect costs will be collected and analyzed using open secondary data, such as the existing Health Insurance Review and Assessment Service customized data and the Korean medical panel data. Moreover, we will evaluate the stability of the incremental cost-effectiveness ratio through a sensitivity analysis from the societal perspective.

### 3.4. Statistical Methods

#### 3.4.1. Sample Size Calculation

This clinical trial aims to evaluate the efficacy and safety of the combined administration of donepezil and WCW for treating BPSD in patients with mild probable AD. According to Okahara et al. [43], the standard deviation of the combination group (intervention group, donepezil + herbal medicine) and single group (control group, donepezil) is estimated to be 13, and the mean changes are 10.2 and 1.4, respectively. Therefore, 33 participants per group are required to obtain a statistical power of at least 80% under the significance level of 5% on both sides, thus generating a total of 66 participants. Considering a 10% dropout rate, we will require 74 participants (37 participants per group) for the study.

Hypothesis:

$$H_{0}:\;\mu_{test}-\mu_{control}=0\;\mathrm{vs}.\;H_{1}:\;\mu_{test}-\mu_{control}\neq 0$$

Estimation formula for the number of participants:

$$n=\frac{2\sigma^{2}(Z_{\frac{\alpha }{2}}+Z_{\beta }\;{)}^{2}}{(\mu_{test}-\mu_{control}{)}^{2}}$$

* *μ_test_*: NPI population mean change at four weeks from baseline in the test group (10.2). * *μ_control_*: NPI population mean change at four weeks from baseline in the control group (1.4). σ: Common standard deviation of the test group and control group (=13) *Z_α_*_/2_ = 1.96, *Z_β_* = 0.842.

#### 3.4.2. Statistical Analysis

This clinical trial will follow the intention-to-treat (ITT) principle, and the data obtained from the participants will be principally divided into the Safety Set, Full Analysis Set (FAS), and Per-Protocol Set (PPS). The core population of this clinical trial will be defined as the PPS; the results of the FAS and PPS will be presented together for the efficacy endpoint. Moreover, the results of the safety endpoint will be presented for the safety group.

The FAS will comprise a group of participants for which at least one value of the primary efficacy endpoint is measured after randomization, following the ITT principle. The PPS will include the following participants from the FAS targets: satisfied the selection criteria, no major protocol violations, a primary efficacy endpoint at 24 weeks following randomization, and with ≥80% medication compliance during the treatment period. The safety group will include the participants who have consumed clinical drugs at least once following randomization.

We will obtain a two-sided 95% confidence interval (one-sided 97.5% confidence interval) for the difference in the altered total NPI score after 0 weeks and 24 weeks between the groups. An upper limit < 0 will confirm the superiority of the combination group to the single group. The confidence intervals will be obtained by a covariance analysis, and the baseline values of the participant’s severity, ADAS-Cog, and MMSE will be used as the covariates.

For all secondary endpoints, a two-sided test will be performed at a significance level of 5% to compare the groups at week 0 or week 1. For continuous variables, such as MMSE, descriptive statistics (observed dimension, mean, standard deviation, median, minimum, maximum, and confidence interval) will be presented for each value per visit (week 0, week 8, week 16, and week 24).

The Student’s *t*-test will be used as the main analysis to compare the variation of week 0 versus week 24 between the two groups, and a Wilcoxon’s rank-sum test will be performed if the normality assumption is not satisfied. Repeated measurement variance analysis will compare the two groups for the amount of change at each visit point.

While analyzing the primary efficacy endpoint, if there are missing values at a certain point or the participant is dropped before terminating the clinical trial, the most recently obtained data will be analyzed as if obtained at that time point (Last Observation Carried Forward Analysis). However, the analysis will be performed using the data (available data set) without substituting the missing values for the laboratory test or other items.

### 3.5. Safety Evaluation

For each adverse event, this study will report on the numbers and percentages of adverse effects related to the drug and participants who experience one or more adverse events. In addition, the number and percentage of participants who report seriousness, severity, relevance to clinical drugs, and results of adverse reactions will be classified by group. Moreover, we will summarize the number and percentage of the reported participants.

For the continuous data, the mean, standard deviation, minimum, and maximum values will be arranged by group. Differences between the groups will be assessed using a two-sample t-test and Wilcoxon’s Rank Sum test for satisfied and unsatisfied normality, respectively. The categorical data will be classified as normal and abnormal, and differences between the groups will be evaluated using the Chi-square test or Fisher’s exact test. Differences before and after drug administration will be analyzed using the McNemar’s test.

### 3.6. Quality Assurance

The person in charge of the clinical research will explain all adverse reactions to the co-investigator, participant, or guardian, besides providing training to report all phenomena that may occur following administration. Any severe adverse reaction during the study will be reported to the Institutional Ethics Review Board (IRB) of the relevant institution to determine the continuation or termination of the study. In the event of a participant suffering any impairment, this study will follow the rules on compensation for the victims submitted to the IRB.

Additional safety information will be periodically reported until the end of the adverse events (the disappearance of an adverse event or the inability to follow up). The principal investigator shall address all matters while implementing the clinical research in accordance with the tenets of the Declaration of Helsinki. Approval from the IRBs of Dongguk University will be obtained upon revising the protocol. According to the regulations, we will obtain approval from the Ministry of Food and Drug Safety if necessary.

### 3.7. Data Management and Monitoring

The collected data will be managed with an electronic case report form using the internet-based clinical research and trial management system (iCReaT) operated by the National Institute of Health, Korea Centers for Disease Control and Prevention (iCReaT study number C210063). A separate company employee designated by the principal investigator will regularly visit the research institute to monitor the research. During the visit, an independent monitor not involved in the clinical trial and not affiliated with the sponsors will assess the original patient records, management records, and data storage (research file). In addition, the monitor will closely review the progress of the study and consult with the researcher upon encountering problems.

### 3.8. Criteria for Clinical Trial Termination

The principal investigator may suspend the trial upon encountering any of the following events. Subsequently, the reason shall be submitted in writing to the IRB.

(1) An unforeseen obvious or unacceptable risk to the participant.
(2) Moderate or severe adverse reactions related to the test drugs occur in >25% of the participants.
(3) The principal investigator decides to suspend or discontinue the trial.

### 3.9. Ethical Statement

This protocol was reviewed and approved by the IRBs of the Dongguk Ilsan Korean Medicine Hospital of the Dongguk University (IRB approval no. DUIOH 2020-03-006) and the Ilsan Medicine Hospital of the Dongguk University (IRB approval no. DUIH 2020-07-002). To maintain confidentiality, access to patient data will be limited to the principal investigators and sub-investigators. The person in charge of the research will retain copies of the medical records and case records related to the research for the period specified in the relevant regulations, based on the Korean Good Clinical Practice guidelines.

Human material will be disposed of appropriately, following all applicable laws and hospital regulations. The participants should agree on allowing the specimens to be preserved for the purpose of analysis in this study or for other research purposes and potentially provided to other researchers, as well as allowing their personal information to be provided along with the specimens.

## 4. Expected Results

This clinical trial will be a randomized, partial-blind, parallel-group study designed to assess the efficacy and safety of WCW in combination with donepezil on BPSD in patients with probable AD.

Herbal medicine is widely used in AD treatment, particularly in East Asian countries. Herbal medicines increase neurogenesis and have anti-inflammatory, anti-apoptotic, and antioxidant actions [8]. Therefore, they can be helpful for neurodegenerative diseases, including dementia. Moreover, the co-administration of herbal and conventional medicine is emerging as a promising strategy for controlling BPSD in patients with dementia [18].

WCW has a cerebral neuroprotective action, thus suggesting its use for neurological and psychiatric disorders related to hippocampal and cerebral cortical damage, including dementia [12]. Previously, researchers have conducted preclinical studies on the efficacy of WCW on memory disorders and behavioral and psychological symptoms. WCW suppressed the deleterious effects of a high-fat diet in mouse models of short- and long-term cognitive deficits [44]. In addition, it improved memory in a mouse model that was administered a NOS inhibitor [45], and exposure to WCW improved depression-like behavioral symptoms in a mouse model of induced stress [15]. Nevertheless, no clinical trials have examined its effects on BPSD control in probable AD. This study will be the first randomized controlled trial to evaluate the efficacy and safety of WCW as a BPSD treatment and will provide the basis for using WCW to treat BPSD in mild probable AD.

In a preclinical experiment, the combined effects of WCW and donepezil were confirmed in an in vivo experiment. First, the effects of the combination were confirmed using a scopolamine-induced ICR mouse model. In the passive avoidance and water maze tests, significant improvements in the memory and cognitive function were observed in the combined administration group compared to the single administration group. In addition, there was no abnormality in the evaluation of liver toxicity. Although the results have not been published, the combined administration of WCW and donepezil was approved by the IRB and the Ministry of Food and Drug Safety based on these results.

In the authors’ clinic, 172 patients have been treated with a combination of donepezil and WCW for more than 28 days in 22 cases and with a maximum frequency of 328 administrations per person. No side effects were reported in any of the patients, and these data were submitted to the Ministry of Food and Drug Safety to obtain permission to conduct the proposed clinical trial. In addition, abnormal reactions will be reported during the study, and laboratory tests will be conducted periodically to monitor the potential occurrence of side effects.

Owing to its multidisciplinary design, we will perform research on human derivatives. The lipid body confirms the change in lipid concentration in diseased cells, tissues, and biological fluids, and it is possible to screen for diseases and discover treatment targets through relatively small levels of molecular changes [46]. First, we will analyze the difference in metabolite and lipid profile in samples (urine, saliva, and serum) from patients with probable AD treated with either a single administration of donepezil or combined administration of donepezil and WCW. This step will enable the identification of a probable AD treatment target biomarker for the combined administration of donepezil and WCW. Second, we will analyze the difference in metabolite and lipid profile in the samples, according to the presence of reactivity to the combined administration of donepezil and WCW. This technique will help establish the biomarkers and models for predicting drug effects that can predict the reactivity to the co-administration of drugs.

A biomarker is an indicator of the presence or severity of a disease. An early diagnosis and prevention of dementia is essential for better treatment and prognosis, and various clinical biomarkers are being proposed to address this issue. Researchers have adopted diverse methods (e.g., genetic, biochemical, and brain imaging markers) to identify biomarkers [47]. A reduced graphene oxide (rGO) biosensor will be utilized for the biomarker measurement in this study. The detection of biomarkers is accomplished by measuring conductance change following antibody-antigen reaction on the rGO surface [48,49]. We will perform antibody immobilization and antigen reaction as follows: Both 6E10 antibody and an antibody of neuroprotective substance will be diluted to a concentration of 10 μg/mL with 0.1X phosphate-buffered saline (PBS) solution. Subsequently, each antibody will be immobilized at the rGO surface with 1-ethyl-3-(3-dimethylaminopropyl) carbodiimide (2 mM) and N-hydroxysuccinimide (NHS, 8 mM) for 150 min. The residual materials will be removed by rinsing the rGO surface with 0.1X PBS and deionized (DI) water. The plasma samples will be diluted in 0.1X PBS (1:5 ratio). The diluted plasma will be dropped on the antibody-immobilized rGO surface and left to react for 30 min. Following the reaction, the surface will be rinsed with 0.1X PBS and DI water.

This trial has some limitations. First, implementing a double-blind design is impossible because of the uniqueness of the WCW formulation. As explained above, WCW is a combination of herbal medicines with a unique taste and scent, and the gold leaf covering on the outside has a soothing effect. It is difficult to make a placebo that is similar to WCW. To overcome this limitation, we have decided to blind the raters instead. Second, the WCW (Kwangdong Pharmaceutical Company, Seocho-gu, Seoul) used in this clinical trial contains civet, which is slightly different from the prescription composition used in existing research studies (contains musk); however, several experimental studies have shown that products containing civet have a similar efficacy to those containing musk [50]. In terms of anti-stress action, WCW containing civet has been reported to be more effective than treatments containing musk [51].

Despite these limitations, the benefits of conducting this study are clear. Overall, this trial will provide evidence for the efficacy and safety of WCW combined with donepezil for BPSD in patients with probable AD.

## Figures and Tables

**Table 1:** Composition of the herbal formula Woohwangchungsimwon.

Herbal Name	Scientific Name	Amount (mg)
Dioscorea Rhizome	*Dioscorea batatas* Decne	263
Glycyrrhiza	*Glycyrrhiza glabra* L.	188
Ginseng	*Panax ginseng* C.A Mey	94
Cat-Tail	*Typha orientalis* C. Presl	94
Massa Medicata Fermentata		94
Glycine Semen Germinatum	*Glycine max* subsp. *soja* (Siebold and Zucc.) H. Ohashi	66
Cinnamon Bark	*Cinnamomum cassia* (Nees and T. Nees) J. Presl	66
Glue	*Equus asinus* L.	66
Peony Root	*Paeonia lactiflora* Pall.	56
Liriope Tuber	*Liriope platyphylla* F.T. Wang and Tang	56
Scutellaria Root	*Scutellaria baicalensis* Georgi	56
Angelica Gigas Root	*Angelica gigas* Nakai	56
Saposhnikovia Root	*Saposhnikovia divaricata* (Turcz.) Schischk	56
Atractylodes Rhizome White	*Atractylis japonica* (Koidz. Ex Kitam.) Kitag.	56
Bupleurum Root	*Bupleurum falcatum* L.	47
Platycodon Root	*Platycodon grandiflorum* A.	47
Apricot Kernel	*Prunus armeniaca* L.	47
Hoelen	*Poria cocos* Wolf	47
Cnidium Rhizome	*Cnidium officinale* Makino	47
Oriental Bezoar	*Bostaurus Linne var. domesticus* Gmelin.	45
Gazelle Horn	*Saiga tatarica* L.	38
Civet	*Viverra zibetha* L.	114
Borneolum	*Dryobalanops aromatica* C.F. Gaertn	38
Ampelopsis Radix	*Ampelopsis japonica* (Thumb.) Makino	28
Ginger	*Zingiber officinale* Roscoe	28
Mel	*Acalypha indica* Radoszkowski	3117
Aurum		quantum satis

**Table 2:** Trial Schedule.

	Study Period				
	Screening	Treatment Period			Closing
Trial Schedule	Visit 1	Visit 2 (Week 0)	Visit 3 (Week 8)	Visit 4 (Week 16)	Visit 5 (Week 24)
Informed consent, assigned screening number, demographics, and medical history	●				
12-lead ECG, Chest X-ray, B-MRI ^†^	●				
Inclusion/Exclusion criteria	●	●			
Randomization		●			
Changed medical history		●	●	●	●
Clinical trial drug administration		●	●	●	
Compliance			●	●	●
K-MMSE ^‡^, NPI ^‡^	●	●	●	●	●
ADAS-cog, SNSB, EQ-5D, Economic evaluation		●			●
GDetS, K-IADL, GQOL, R-MBPC		●	●		●
CDR ^‡^, BMI	●	●	●		●
PSQI, GDepS, STAI		●	●	●	●
Pattern Identification, TCI, and Sa-sang	●				
Human derivatives study (urine, saliva)	●	●	●	●	●
fMRI (n = 20)	●				●
Physical examination, vital signs	●	●	●	●	●
Laboratory test ^§^	●		●		●
Adverse reactions			●	●	●
Blinding maintenance test					●

●, check on visit; ADAS-cog, Alzheimer’s Disease Assessment Scale-Cognitive Subscale; BMI, Body Mass Index; B-MRI, Brain Magnetic Resonance Imaging; CDR, Clinical Dementia Rating; EQ-5D, Euro-Quality of Life-5 Dimension; fMRI, Functional Magnetic Resonance Imaging; GQOL, Geriatric Quality of Life scale; K-IADL, Korean version of Instrumental Activities of Daily Living; K-MMSE, Korean version of the Mini-Mental State Examination; NPI, Neuro-Psychiatric Inventory; PSQI, Pittsburgh Sleep Quality Index; R-MBPC, Revised-Memory and Behavior Problem Checklist; SNSB, Seoul Neuropsychologic Screening Battery; STAI, State-Trait Anxiety Inventory; and TCI, Temperament, and Character Inventory. ^†^ B-MRI performed at the screening can be replaced with the results within one year from the screening date. ^‡^ K-MMSE, NPI, and CDR performed at the screening can be replaced with the results within six months. ^§^ Clinical pathology tests performed at the screening can be replaced with the results within six months of screening.

**Table 3:** Eligibility criteria.

Inclusion Criteria	Exclusion Criteria
Men and women aged ≥50 years diagnosed with Alzheimer’s disease by a specialist according to the DSM-5 and NINCDS-ADRDA standards. K-MMSE: participants with a score ranging from 10 to 26 at the screening visit. CDR: 0.5–1. NPI: at least one symptom score ≥4 on the substructure scale. Participants under tranquilizers or sleeping pills for four weeks can continue them, but the dose should not be increased, nor should they begin afresh. Quetiapine or risperidone can be used upon the worsening of BPSD, but the prescribed dose or duration should be maintained at a minimum ^†^. HIS: ≤4. Participants under 5 mg of donepezil (acetylcholine esterase inhibitor (AchEI)) for more than four weeks and are maintaining a stable state without side effects after consuming the maintenance dose. (including those under brain function-improving drugs (e.g., gliatilin, gliatamin) and cerebral blood-flow-improving drugs (e.g., ginexin and tanamine).) Participants maintained in a stable state without changes in the drugs consumed for the underlying disease for more than two weeks and are scheduled for stable administration in the trial period. Participants who live with a guardian who understands their requirements according to the procedure specified in the trial protocol and who can visit the center with their guardian. Participants who have signed a written consent form after being explained the purpose, method, and effects of the trial.	Participants with dementia owing to causes other than Alzheimer’s disease: degenerative brain diseases, such as vascular dementia, Parkinson’s disease, and Huntington’s disease. Participants with general conditions that cause dementia, such as hypothyroidism, vitamin B12 or folate deficiency, niacin deficiency, hyperkalemia, neurosyphilis, and human immunodeficiency virus disease. Participants with clear clinical evidence of cerebrovascular disease or suspected territory infarct of cerebrovascular disease owing to multiple strokes assessed by MRI. Participants with a history of neurological disorders, such as epilepsy, local brain injury, head trauma, or stroke. Participants with a history of major psychiatric disorders, such as schizophrenia, delusional disorder, depression, bipolar disorder, or alcohol or substance abuse disorder, diagnosed by DSM-5. Participants currently under psychotropic drugs or hormones. Participants with severely unstable medical conditions (according to the judgment of the doctor in charge, based on the results of the clinical laboratory tests, ECG, chest PA, and vital signs). Hypertensive patients with systolic blood pressure > 165 mmHg and diastolic blood pressure > 96 mmHg. Patients with diabetes uncontrolled by hypoglycemic drugs or those with insulin-dependent diabetes. Participants with a clinically significant liver disease or LFT indicator levels exceeding twice the upper limit of normal. Participants with chronic renal failure or >1.5 times the normal upper limit of serum creatinine. Participants with gastrointestinal, endocrine, and cardiovascular diseases that are not controlled by diet or medications. Participants with a disease that may affect the absorption of drugs or lead to digestive problems after undergoing a related surgery. Participants with hypersensitive reactions or allergies to the components of the trial drugs. Women of childbearing age having the following conditions: − Pregnancy confirmed with serum or urine samples − Lactating − Planning a pregnancy − Not using recognized contraception methods (e.g., sterilization, intrauterine contraceptive devices, condoms, contraceptive creams, diaphragms with jelly, or foam. Hormone preparations cannot be used). Participants who have partaken in other clinical trials within three months before the screening of this trial. Participants who do not understand the consent form owing to mental retardation, emotional or intellectual problems, or have difficulty following the study. Participants who are judged unsuitable for the trial by the investigator.

BPSD, Behavioral and Psychological Symptoms of Dementia; CDR, Clinical Dementia Rating; chest PA, chest X-ray posteroanterior; DSM-5, Diagnostic and Statistical Manual of Mental Disorders-5; ECG, Electrocardiogram; HIS, Hachinski Ischemic Score; K-MMSE, Korean version of the Mini-Mental State Examination; LFT, Liver function tests; MRI, Magnetic Resonance Imaging; NINCDS-ADRDA, National Institute of Neurological and Communicative Disorders and Stroke and the Alzheimer’s Disease and Related Disorders Association; NPI, Neuro-Psychiatric Inventory. ^†^ When administering quetiapine or risperidone, the plan is to administer a low dose while closely monitoring adverse events, such as sedation and extrapyramidal symptoms. The occurrence and severity of the adverse event will be evaluated and recorded at each patient visit, and when an adverse event occurs, the dose will be reduced, or the medication will be discontinued according to the clinician’s judgment.

## Data Availability

The data that support the findings of this protocol shall be available from the corresponding author upon reasonable request.

## Trial Status

Recruitment began on 21 December 2020. The current version of the protocol is 2.1, published on 19 August 2021. We expect the recruitment to be completed by 27 February 2024. The trial was registered at the Clinical Research Information Service (CRIS) on 10 December 2020 (KCT0005669).
